# Behavioral Plasticity of Rewilding Milu in Mountainous Region of Northern China

**DOI:** 10.3390/ani15202993

**Published:** 2025-10-15

**Authors:** Jialiang Ma, Jiade Bai, Ritu Su, Haibo Ma, Chenmiao Feng, Zhenyu Zhong, Qingyun Guo, Qingxun Zhang, Zhibin Cheng, Kun Cheng

**Affiliations:** 1 College of Wildlife and Protected Area, Northeast Forestry University, Harbin 150040, China; 13026556767@163.com; 2 Beijing Milu Ecological Research Center, Beijing 100076, China; baijiade234@aliyun.com (J.B.); fengmiao1988@163.com (C.F.); miluhome@126.com (Z.Z.); guoqingyun1987@126.com (Q.G.); zhangqingxun1990@126.com (Q.Z.); 3 Beijing Biodiversity Conservation Research Center, Beijing 100076, China; 4 Inner Mongolia Daqingshan National Nature Reserve Management Bureau, Huhehaote 010010, China; 13804743238@163.com (R.S.); zbx6621mhh@163.com (H.M.)

**Keywords:** *Elaphurus davidianus*, rewilding, home range, activity rhythm, movement distance, extreme environment, sexual differences

## Abstract

**Simple Summary:**

There is limited research on the behavioral adaptability of rewilding endangered species into relatively harsh environments. Historically, Milu (*Elaphurus davidianus*) primarily inhabited warm, humid low-altitude plains and wetlands. In 2021, 27 Milu were introduced into the mid-altitude Daqing Mountains of Inner Mongolia. This study aims to reveal the behavioral adaptability of Milu under harsh environmental conditions. The results indicated that after rewilding into the Mongolian Daqing Mountains, the Milu exhibited strong behavioral plasticity in terms of home range size, movement distance, and circadian rhythms.

**Abstract:**

Species rewilding, as a key strategy for rescuing endangered species and rebuilding wild populations, fundamentally relies on the behavioral plasticity of specific wildlife species. Although most current rewilding initiatives select optimal habitats, research on behavioral adaptation mechanisms in more challenging, extreme environments remains lacking. The Milu (*Elaphurus davidianus*), a typical wetland and plain species, naturally inhabits the warm marshlands of the Yangtze and Yellow River basins. In this study, using GPS tracking data, we focused on a population of rewilding Milu on the Inner Mongolia Plateau to investigate behavioral plasticity in terms of home range area, activity rhythm, and movement distance, aimed to elucidate their survival adaptation strategies within mid-elevation and cold environments. The results indicated significant seasonal and sex-based differences in both home range and movement distance: home ranges contract and movement distances are minimized during winter, while spatial activity expands markedly in summer—and continues to increase year by year following rewilding. During the study period, the number of daily activity peaks per individual ranged from zero to four. Furthermore, peak timing exhibited clear seasonal variation, with crepuscular patterns—morning and evening activity peaks—predominant across most months. Approximately three months after release, the activity rhythms of both males and females stabilized. These findings reveal key behavioral adjustments of Milu translocated to a mountainous, cold-temperate environment outside its original distribution range, and provide a scientific basis for long-term management and for assessing the ecological adaptability of this introduced population.

## 1. Introduction

In recent years, behavioral plasticity has emerged as a crucial adaptive strategy through which animals respond to environmental change, drawing significant attention from the fields of ecology, behavioral science, and evolutionary biology [1,2,3]. This term refers to an animal’s ability to adjust its behavior in response to environmental fluctuations, individual needs, or social interactions [4]. Research into behavioral plasticity—how animals modify their behavioral patterns in response to changing environments, resource variability, or social dynamics—not only advances our understanding of ecological adaptation mechanisms but also provides critical insights into predicting biodiversity trends under the influence of human activity and climate change [5,6].

Numerous studies have demonstrated widespread behavioral plasticity across diverse animal taxa [7,8]. A striking example comes from an analysis of 89,000 mammalian activity records, which revealed that while nearly half of the studied species retained traditional diurnal or nocturnal routines, many others exhibited pronounced diel plasticity in response to human disturbance [4]. This plasticity is particularly nuanced in migratory behaviors of ungulates. Research encompassing 27 species—such as caribou (*Rangifer tarandus*), wapiti (*Cervus canadensis*), and mule deer (*Odocoileus hemionus*)—has demonstrated their widespread plasticity in migration timing, patterns, and routes in response to environmental changes like climate change and anthropogenic pressures [9]. Beyond migration, plasticity extends to other behavioral domains. For instance, white-tailed deer (*Odocoileus virginianus*) compensate for thermal stress by reducing daytime feeding and increasing nocturnal foraging to meet energetic requirements [10]. In cervids, this adaptability can be more pronounced: female wapiti dynamically adjust migratory routes and timing in response to interannual fluctuations in vegetation availability and predation risk [11]. Similarly, white-tailed deer modulate social strategies, such as group size and vigilance levels, based on perceived predation threat [12]. However, behavioral plasticity can entail unforeseen ecological trade-offs. A case in point is the Eurasian moose (*Alces alces*), which shifts to higher elevations to escape winter tick (*Dermacentor albipictus*) infestations. While this behavioral shift reduces tick loads, it concurrently compromises body condition due to poorer forage quality at higher altitudes [13]. Collectively, these findings underscore that shifts in space use in response to seasonal climatic variation are a common adaptive strategy, enabling animals to align their movements with temporal changes in resource availability and habitat conditions [14]. Rewilding, is a potent strategy for restoring populations of endangered species [15,16], yet it remains a high-risk endeavor whose success is often constrained by a spectrum of interconnected challenges. These can be broadly categorized into ecological, social, and species-specific risks. Ecological and anthropogenic pressures, such as habitat degradation, climatic mismatch, and Human–Wildlife conflict, are evident in cases like the Arabian oryx (*Oryx leucoryx*) in Oman, where populations were limited by drought and illegal hunting [17,18,19,20,21,22]. Reintroduced species must also navigate natural ecological pressures, such as the selective predation by wolves on rewilding American bison (*Bison bison*) [23]. Perhaps the most nuanced challenges are socio-economic factors, exemplified by the significant community resistance to African lion recovery in Zambia due to livestock predation, inequitable benefits, and governance issues [24]. Finally, the success of establishing resilient populations is threatened by inherent species-specific limitations, including behavioral inflexibility, disease, and genetic risks [17,18,19,20,21]. Consequently, post-release monitoring and studies of adaptive responses are essential components of rewilding programs [25,26,27]. However, research on behavioral plasticity in rewilding species remains scarce.

The Milu (Père David’s deer, *Elaphurus davidianus*) is a rare deer endemic to China [28]. Historically extinct in the wild, it has recovered gradually through captive breeding and reintroduction efforts [29]. Originally, Milu inhabited the warm, moist, floodplain wetlands of the Yangtze and Yellow River basins [30]. Since 1998, conservation efforts have focused on rewilding programs aimed at reestablishing self-sustaining populations within its historical range, including the Dongting and Poyang Lakes in the Yangtze Basin and the intertidal wetlands along the Yellow Sea coast [29,31,32]. Several studies have monitored the behavior of rewilding Milu using GPS tracking. In the Yellow Sea coastal wetland, female Milu showed pronounced seasonal shifts in home range size influenced by human activity and food availability—expanding into farmland during winter and spring, and contracting to tidal meadows and reedbeds within protected zones in summer and autumn [31]. Similarly, in the Dongting Lake region, Milu had significantly larger winter home ranges than in summer [33]. Ambient temperature further influenced their daily activity pattern: Milu at Dongting Lake rested mostly before 09:00 and after 18:00, with a foraging peak at 14:00, and exhibited sharply increased vigilance following disturbances [34].

Milu have been successfully reintroduced in nine field release events within China’s Yangtze River basin and Huaihai tidal wetlands [29], where are all the historical range with the highest archaeological fossil occurrences of this species [35,36]. Milu was also introduced to other areas outside its historical distribution range, such as Inner Mongolia’s Daqingshan in 2021. Compared with warm and humid lowland swamp habitats, the mid-altitude Mongolian Plateau is characterized by a typical temperate continental climate, complex topography, and harsher environmental conditions. These extreme environmental factors present significant challenges to Milu’s survival. Therefore, investigating the behavioral adaptation mechanisms—specifically changes in activity rhythm, home range and movement characteristics of Milu in mountainous region is crucial for understanding their survival strategies and assessing the adaptive capacity of these rewilding populations.

## 2. Materials and Methods

### 2.1. Study Area

In order to establish more populations of Milu and thus increase the chances of conservation, it has been carried out a rewilding program in the Daqingshan Nature Reserve, which is located in the Yinshan Mountains of central-western Inner Mongolia and spanning parts of Hohhot, Baotou, and Ulanqab. The reserve stretches approximately 217 km from east to west, with an average north–south width of about 18 km, covering a total area of 388,900 ha, making it the largest forest-ecosystem nature reserve in northern China. Daqingshan, located on the southeastern margin of the Mongolian Plateau, borders the Yellow River Plain to the south, where Milu was once widely distributed historically. Located in the mid-temperate zone with a typical continental climate, the reserve has an average elevation of approximately 1850 m, with its highest peak reaching 2338 m. The annual average temperature ranges between 3 °C and 5 °C, and the annual precipitation is approximately 320–450 mm. Prior to program implementation, we conducted feasibility analyses taking into account among other factors, such as water and food resources, shelter, and human interference.

**Figure 1:**
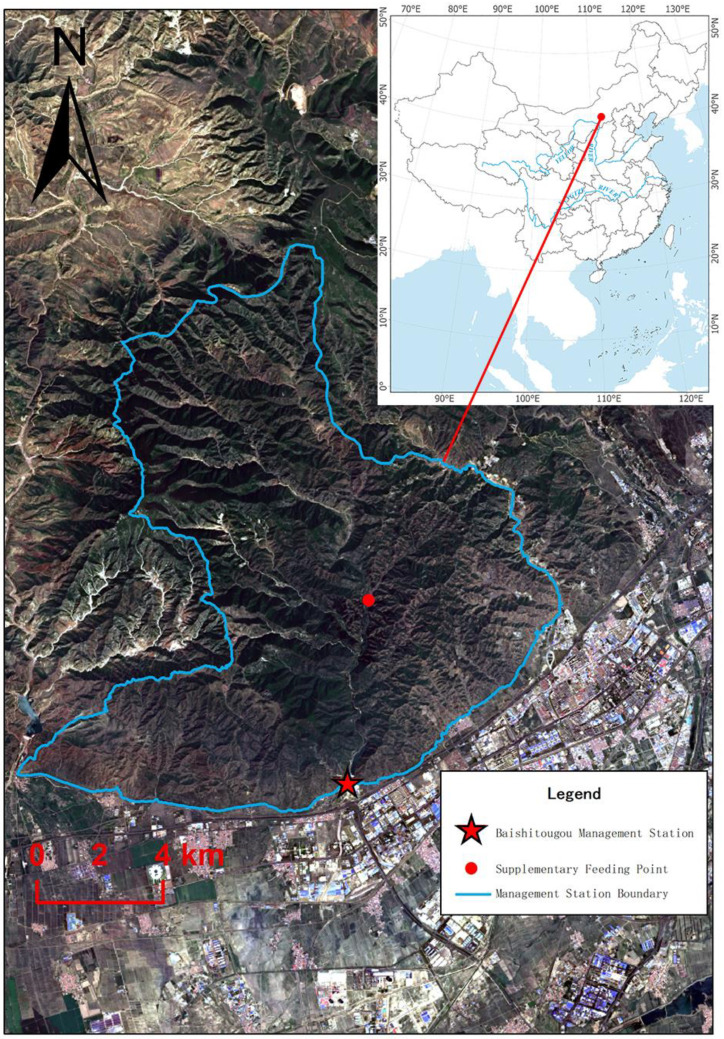
Location map of the rewilding site (image source: https://earthexplorer.usgs.gov/ (accessed on 15 August 2025)).

The rewilding site for the Milu in this study is situated in the buffer zone at Baishitougou Management Station (111.468683° E, 40.822307° N) of Daqingshan Nature Reserve (Figure 1). The management station is primarily located in a natural secondary forest area, featuring multiple natural springs and perennial unfrozen streams. The main vegetation includes trees, shrubs, and herbaceous plants such as: Chinese pine (*Pinus tabuliformis*), White birch (*Betula platyphylla*), Dahurian larch (*Larix gmelinii*), Juniper (*Juniperus rigida*), Mongolian linden (*Tilia mongolica*), Siberian apricot (*Prunus sibirica*), David poplar (*Populus davidiana*), Mountain elm (*Ulmus glaucescens*), Hazelnut (*Corylus heterophylla*), Meadow sweet (*Spiraea salicifolia*), Yellow rose (*Rosa xanthina*), Feather grass (*Stipa capillata*), Chinese leygrass (*Leymus chinensis*), Lambsquarters (*Chenopodium album*). The winter minimum temperature can drop to −30 °C. This site is in the reserve’s buffer zone and lies in a remote, uninhabited area with virtually no human disturbance, making it an ideal location for studying the deer’s autonomous adaptation behaviors in extreme environments. There was an iron fence barrier (111.454377° E, 40.803294° N) in the south preventing the Milu from spreading south into the urban area of Hohhot.

### 2.2. Animals and Data Collection

27 milu were translocated to Daqingshan from Nanhaizi, Beijing, and Dafeng, Jiangsu Province. Prior to rewilding, GPS satellite-tracking collars (HQAN40L, 800 g, 5-year battery life, solar-powered, Hunan Global Messenger Technology Co., Ltd., Changsha, China) were fitted on 11 Milu (3 males and 8 females). To minimize the impact of collaring on natural behavior, the deer were anesthetized one month before release, and the collars were installed to ensure adequate acclimation time before data collection [37]. These GPS collars were equipped with tri-axial accelerometer sensors to continuously record activity data [37,38]. The sensors monitor real-time acceleration on the X, Y, and Z axes, with a default sensitivity threshold of 0.15 g (gravitational acceleration). Whenever acceleration on any axis exceeded this threshold, the device recorded an additional step, thereby quantifying the deer’s activity level. The GPS location and activity information were automatically saved every two hours. This data was then retrieved using Hunan Global Messenger’s satellite tracking software (version 3.0401). In addition, the collars data was used to monitor and analyze home range and movement behavior of the deer population.

Seasons were delineated based on local climatic conditions: spring (March to May), summer (June to August), autumn (September to November), and winter (December to February). Due to the limited effective collar time for some individuals and the absence of data for September 2021, average home-range areas and movement distances for both sexes were calculated using data from four seasons only: 2021 autumn (October–November 2021), 2021 winter (December 2021–February 2022), 2022 spring (March–May 2022), and 2022 summer (June–August 2022). The average diurnal activity rhythms for both sexes span from October 2021 to September 2022, covering a total of 12 months. We provided supplementary feed (hay and concentrated feed) once a year from November to April of the following year. Two individuals—F7 and M10—still have active collars. We analyzed their activity intensity heatmaps, which is a form of data visualization that represents the spatial distribution of an animal’s movement path and frequency, over 14 seasons, from autumn 2021 to winter 2024 and examined differences in diel activity rhythms over 41 months (October 2021 to February 2025).

### 2.3. Data Analysis

All data were compiled and summarized using Excel 2021, and the results are presented as mean ± standard error (SE). The circadian rhythm graphs were produced with GraphPad Prism 5. Home range data (kernel density estimator (KDE), isopleth 95%), distances from the release site, and activity intensity heatmaps were obtained from the v3.0401 Hunan Global Messenger Technology Satellite Tracking Data Management System, which employed a Gaussian kernel function for activity intensity heatmap generation. Satellite images obtained from https://earthexplorer.usgs.gov/ (accessed on 15 August 2025). Additionally, we used ArcGIS Desktop (version 10.8) to create the maps.

Statistical analyses were performed as follows. First, to analyze the sexual and seasonal differences in circadian activity rhythms, we used circular statistics in R (version 4.4.1), specifically Watson’s one-sample and two-sample tests, to assess the significance of the rhythm patterns. Second, sexual differences in the 95% KDE, 50% KDE home range areas, and the farthest movement distances within each season were compared using *t*-tests or Wilcoxon rank-sum tests in R, depending on whether the data met the assumption of normality. Finally, after confirming normality, paired-samples *t*-tests in IBM SPSS Statistics (version 22) were employed to examine seasonal variations in home range and movement distance. Significant differences were considered at *p* < 0.05.

## 3. Results

Of the 27 introduced Milu, over the past four years, two individuals died from an accident and succumbed to a gastrointestinal illness. All other individuals had survived, and the population had now increased to 64, indicating that the rewilding Milu population had successfully adapted to the local climatic conditions (Table 1).

### 3.1. Home Range Area Analysis

**Figure 2:**
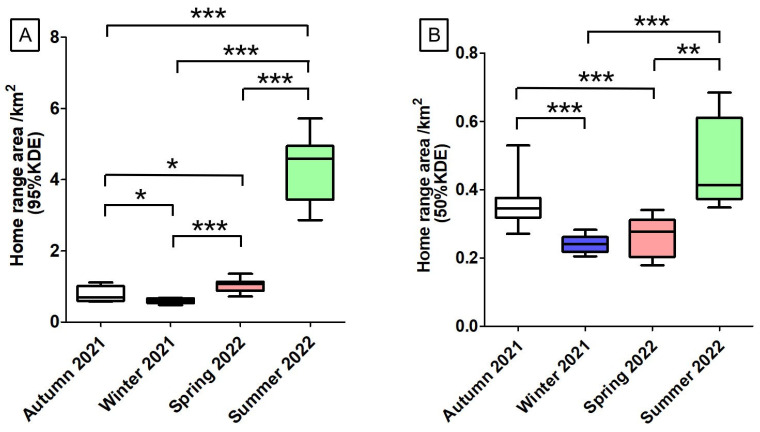
Seasonal home range area of rewilding male and female Milu in Daqingshan ((**A**) is 95%KDE, (**B**) is 50%KDE. Statistically significant differences between seasons: * *p* < 0.05, ** *p* < 0.01, *** *p* < 0.001).

**Figure 3:**
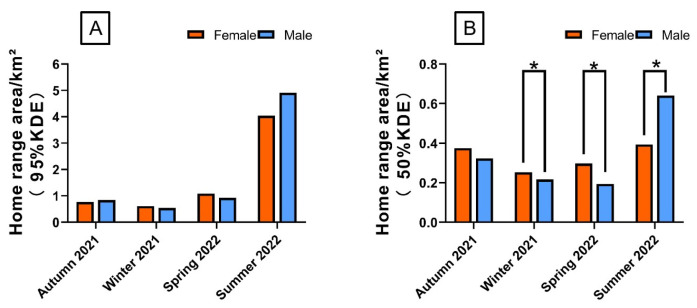
Seasonal home range area of rewilding male and female Milu in the Daqingshan ((**A**) is 95%KDE, (**B**) is 50%KDE. Statistically significant differences between sexes: * *p* < 0.05)).

During autumn 2021, winter 2021, spring 2022, and summer 2022, for these 10 Milu deer, there were significant differences in home range area (95% KDE) among the four seasons (*p* > 0.05) (Figure 2A). The male home-range areas (95% KDE) were 0.84 ± 0.06 km^2^, 0.54 ± 0.002 km^2^, 0.92 ± 0.02 km^2^, and 4.91 ± 0.06 km^2^. The corresponding values for females were 0.77 ± 0.04 km^2^, 0.61 ± 0.005 km^2^, 1.08 ± 0.04 km^2^, and 4.04 ± 0.96 km^2^, respectively (Figure 3A). Across all four seasons, the differences in home-range area between sexes were not statistically significant (Mann–Whitney U test, *p* > 0.05).

During autumn 2021, winter 2021, spring 2022, and summer 2022, for these 10 Milu deer, home range areas (50% KDE) varied significantly across seasons (*p* > 0.05), with the exception of no statistically significant differences between autumn 2021 and summer 2022 (*t* = −2.052, *p* = 0.068), as well as between winter 2021 and spring 2022 (*t* = −1.750, *p* = 0.111) (Figure 2B). The male home-range areas (50% KDE) were 0.32 ± 0.02 km^2^, 0.22 ± 0.007 km^2^, 0.19 ± 0.01 km^2^, and 0.64 ± 0.04 km^2^. The corresponding values for females were 0.38 ± 0.07 km^2^, 0.25 ± 0.02 km^2^, 0.3 ± 0.03 km^2^, and 0.39 ± 0.04 km^2^, respectively (Figure 3B). Except for autumn (U = 17.0, *p* = 0.358), significant differences between sexes were found in winter 2021 (*t* = 3.42, *p* = 0.019), spring 2022 (*t* = 8.321, *p* = 0.000), and summer 2022 (*t* = −9.365, *p* = 0.003).

**Figure 4:**
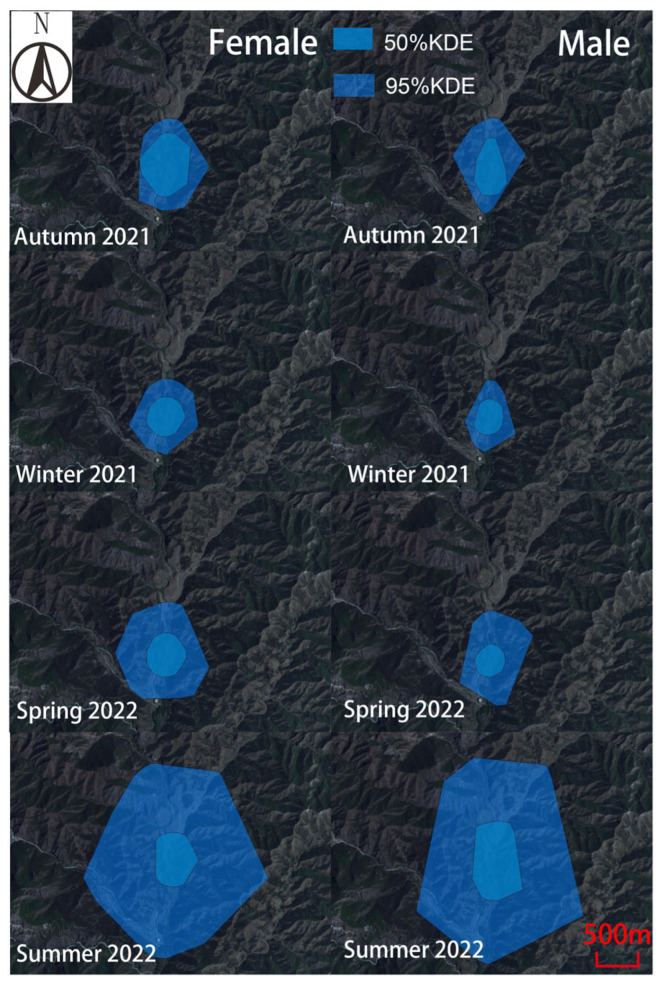
Home range of rewilding male and female Milu from Autumn 2021 to Summer 2022.

**Figure 5:**
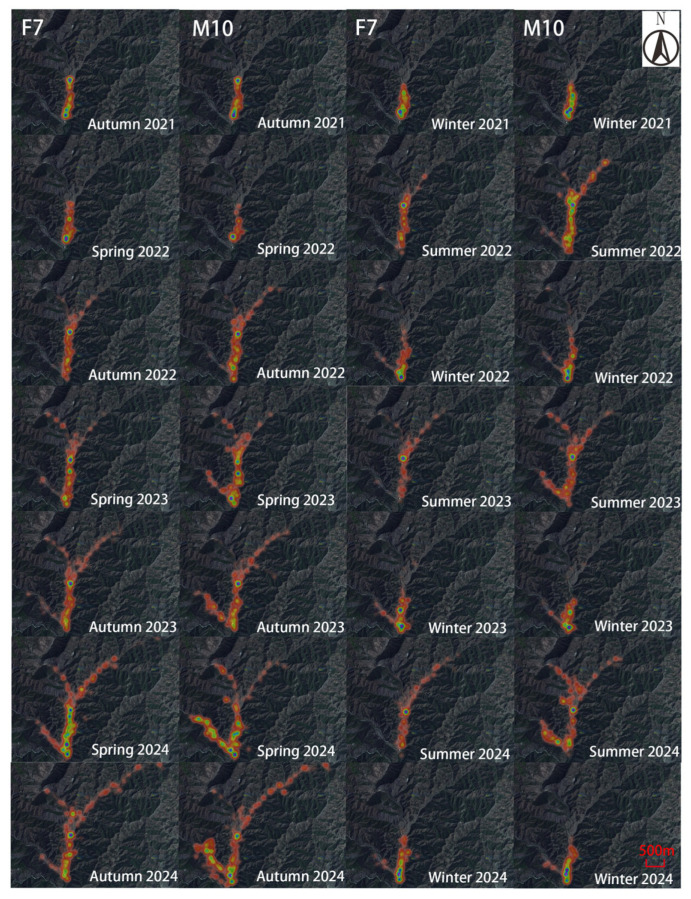
Activity intensity heatmaps of Milu F7 and M10 from Autumn 2021 to Winter 2024 in Daqingshan.

The Milu exhibited the largest activity range during the summer of 2022 (Figure 2, Figure 3 and Figure 4). The individuals F7 and M10 showed pronounced seasonal differences in their activity intensity heatmaps (Figure 5). Combined with daily monitoring observations, the results indicate that although the Milu’s activity range was smallest in winter, the range expanded year over year in spring, summer, and autumn following the rewilding, resulting in a continuous enlargement of their activity range over time (Figure 5).

### 3.2. Movement Distance Analysis

**Figure 6:**
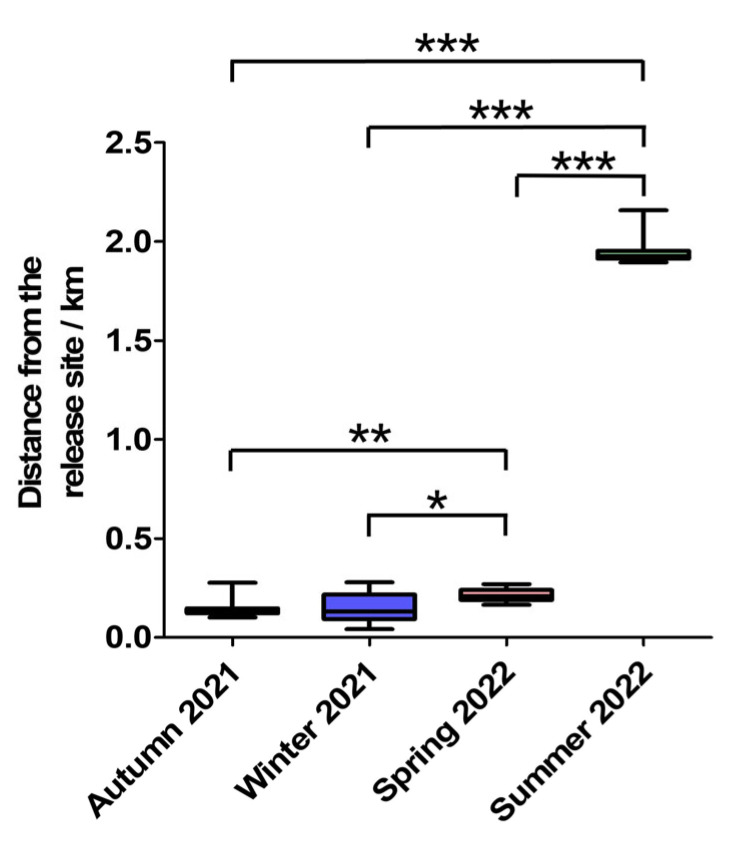
Seasonal movement distances of rewilding Milu in the Daqingshan (* *p* < 0.05, ** *p* < 0.01, *** *p* < 0.001).

During autumn 2021, winter 2021, spring 2022, and summer 2022, the mean movement distances for male Milu were 115.45 ± 12.50 m, 106.08 ± 71.64 m, 225.43 ± 54.41 m, and 1992.99 ± 144.49 m, respectively. For females, the corresponding values were 164.50 ± 52.83 m, 166.41 ± 5536.22 m, 204.41 ± 19.69 m, and 1932.11 ± 18.78 m (Figure 6). A statistically significant difference in movement distance between sexes was observed only in Autumn 2021 (U = 22.0, *p* = 0.0485). For these 10 Milu deer, there were significant differences in movement distances among the four seasons, except between autumn 2021 and winter 2021 (*t* = 0.058, *p* = 0.955). Although the Milu’s movement distance was smallest in winter, the movement distance gradually increased during autumn from 2021 to 2024, while the movement distance in spring and summer also increased year by year starting from 2022 to 2024.

### 3.3. Activity Rhythms Analysis

**Figure 7:**
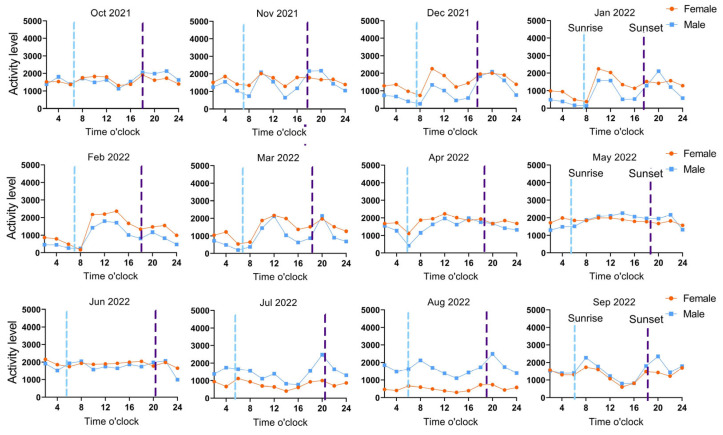
Diurnal activity rhythms of rewilding Milu in the Daqingshan across different months. (The blue dashed line represents sunrise time, and the purple dashed line represents sunset time on the 15th of every month).

The Milu’s activity rhythms exhibit between zero (effectively resulting in zero observable cyclic patterns) and four peak periods (Figure 7), and Watson’s two-sample statistical analysis indicated significant differences in the diurnal activity rhythms between sexes from October 2021 to September 2022 (*p* < 0.01). In October 2021 and from April to June 2022, female Milu had effectively resulted in zero observable cyclic patterns in activity peaks. From December 2021 to March 2022, activity levels of both male and female Milu during nighttime were significantly lower than during daytime. From July to September 2022, both males and females displayed two main activity peaks around 08:00 and 20:00, while from December 2021 to March 2022, their peaks occurred around 10:00 and 22:00. During the first three months after release (October to December 2021), there was a clear difference in activity rhythms between sexes: males exhibited 2–4 peaks (at 04:00, 10:00, 16:00, and 22:00), whereas females showed 1–2 peaks (at 10:00 and 20:00). From January to September 2022, although male activity was notably higher than female activity during July to September 2022, the activity rhythms and peak patterns of both sexes remained closely aligned. From Autumn 2021 to Summer 2022, both male and female Milu exhibited significant activity peaks in all four seasons (*p* < 0.01). Except for the winter of 2021 when the difference in activity rhythms between sexes was significant (U^2^= 0.584, *p* = 0.028), it was not significant in the other three seasons (*p* > 0.05).

Apart from November 2021 and July–September 2022—when the activity rhythm curves of individuals F7 and M10 differ markedly—for the other 37 months, the trends in their activity curves are consistently similar. Observations over 41 consecutive months indicate that during December to March, both F7 and M10 exhibit low activity levels at night (00:00–08:00), with increased activity during daytime hours (10:00–20:00). Except for a pronounced activity peak in F7 during July–September 2022, the activity peaks from April to November are not distinct.

## 4. Discussion

According to theory, the accumulation of knowledge triggers subsequent changes in animal movement behavior; hence, movement behavior can serve as a key indicator of rewilding progress [39]. In this study, we collected 110,773 valid location records from 11 Milu and investigated home-range areas, activity rhythms, and movement distances of the Milu released in Daqingshan. This is the first study to reveal the behavioral adaptive strategies of a rewilding endangered herbivore that originally inhabited flat, temperate regions when exposed to the harsh winter conditions of mid-altitude mountainous environments.

### 4.1. Seasonal Variation in Home Range Patterns of the Rewilding Milu

In cervid species, home-range areas vary significantly by sex, season, and species, driven by a complex interplay of ecological, social, and anthropogenic factors. In terms of sex, males typically have larger home ranges than females—especially during the breeding season. A comprehensive review of red deer biology confirms that these variations are a central aspect of their spatial ecology, with males consistently exhibiting larger home ranges than females—a pattern particularly pronounced during the breeding season [40]. For example, male red deer (*Cervus elaphus*) can have home ranges exceeding 200 km^2^, while females occupy only 50–150 km^2^ [41]; this sexual disparity is further illustrated by a synthesis of data from Western Carpathian populations, where male seasonal ranges significantly surpassed those of females [42]. Similarly, male white-tailed deer expand their ranges to 5–10 km^2^ during the rut, whereas females remain stable at 0.5–3 km^2^ [43]. These sex differences are primarily driven by the physiological needs of males for larger ranges, particularly during the rut. Recent molecular evidence from red deer suggests that such behavioral shifts may be associated with plasticity in gene expression within neuroendocrine pathways [44]. Conversely, females exhibit social plasticity, modulating group size and cohesion in response to predation risk [45], which directly influences their home-range characteristics and habitat selection. Across species, larger cervids such as red deer and moose have significantly larger ranges than smaller species like roe deer (*Capreolus capreolus*, 0.5–2 km^2^) and sika deer (*Cervus nippon*, 1–5 km^2^) [46]. Migratory caribou may have annual home ranges reaching 5000–10,000 km^2^ [47]. Influencing factors include anthropogenic factors (habitat fragmentation [41], urbanization [48], and supplemental feeding [46]) as well as natural factors (food resource distribution [47,49] and predation pressure [41]).

Seasonal variation in home-range size typically follows two patterns. Most cervids—for example, North American wapiti (winter 5–10 km^2^, summer 15–30 km^2^) and Eurasian moose (winter 10–50 km^2^, summer 50–200 km^2^)—tend to show reduced home ranges in winter [41,49]. By contrast, existing studies indicate that Milu often exhibit larger home ranges in winter and smaller ranges in summer. For example, the Dongting Lake population had a winter home range of 187.39 km^2^ versus 14.34 km^2^ in summer [50], and the Yellow Sea tidal-flat population showed 28.18 km^2^ in winter compared with 3.49 km^2^ in summer [31], patterns likely driven by water-level fluctuations and associated changes in food distribution. In this study, we found that Milu in Daqingshan had smaller winter home ranges and larger summer home ranges, likely due to reliance on supplementary feeding in winter leading to food aggregation [51], and because males expand home ranges during the rut in summer in search of mates. This contrast reflects the spatial-temporal heterogeneity of resources across regions—for instance, in Poland, moose summer home ranges (9.0 km^2^) are smaller than winter ones (15.9 km^2^) [52]. Sex differences are mainly due to males requiring larger movement ranges because of their body size and physiological needs [53], whereas females need stable, high-quality food sources for reproduction and rearing. These findings indicate that cervid home-range dynamics are the result of interacting factors including resource distribution, breeding behavior, and human management [54], which align with the results of our study.

### 4.2. Plasticity in Movement Characteristics of the Rewilding Milu

Reintroduced animals released into unfamiliar environments must explore their surroundings to acquire essential survival knowledge [39]. Dispersal behavior post-release varies among species. In the Petit Luberon State Forest in southern France, rewilding roe deer dispersed minimally, remaining largely near the release site—75% of individuals stayed within 4 km—likely due to favorable terrain and low human disturbance [55]. Among white-tailed deer in lower latitudes, 33 % of adult males adopted a “mobile strategy,” with activity ranges averaging 65 km^2^—far exceeding the 3.6 km^2^ of resident individuals—especially during the breeding season [56]. In our study, movement distances increased over time: individuals F7 and M10 extended their distances from release site annually, reaching up to 5 km. This indicates gradual acclimatization to the local climate and expanding range. In contrast, Milu in the Dongting Lake plain and wetlands dispersed up to 20 km—vastly exceeding the movement distance observed in Daqingshan [57].

Based on the “exploration-exploitation trade-off” theory, some scholars have proposed using site fidelity to the release point as a behavioral indicator. Reintroduced individuals transition from an initial phase of slow exploration around the release area to a dual-phase strategy of “large-scale linear movement + core-area intensive activity,” coupled with periodic returns to fixed zones [39]. Translocated wild boars restricted their movements and began to forage more efficiently when released in high-quality habitats rich in bottomland hardwoods. Conversely, individuals introduced to lower-quality environments exhibited a progressive selection for preferred habitat types as their spatial knowledge increased [58]. Similarly, translocated wapiti also exhibited site fidelity, with their movement distance being closely related to the local forage quality [59]. In our study, Milu showed strong site fidelity to areas near the release site. Although the winter conditions at the Baishitougou management station in Daqingshan were extremely harsh—cold and lacking fresh grass—the canyons offered unrestricted movement and relatively abundant edible herbs. Despite this, the deer still preferred to return to the release site for human-provisioned forage. GPS trajectory analysis revealed that even during the summer when fresh tender grass was plentiful, the animals frequently lingered near the release site—perhaps because they felt more secure in a familiar environment. Foraging plasticity involves critical trade-offs. As seen in moose, avoidance of parasitism can lead to nutritional deficits [13]. Furthermore, mule deer adjust their fine-scale movements not just to resource availability, but to its spatiotemporal predictability, indicating a sophisticated energy-balance strategy that operates within their habitats [60].

Research demonstrates that, overall, male cervids tend to disperse over greater distances than females [61,62], consistent with the gene flow patterns in most polygynous ungulates: males promote genetic exchange between populations through long-distance movements, whereas females typically remain near their natal site to reinforce matrilineal bonds. In cervids such as roe deer, males are more inclined to disperse and possess greater dispersal capacity than females [61,62]. A study of 158 North American wapiti revealed that nearly all long-distance dispersers were males, with maximum distances reaching 98 km [62]. Similarly, research on the naturally rewilded Milu population in the Dongting Lake region showed that male groups dispersed more frequently than female and mixed groups; 50% of male dispersal groups comprised only a single adult male, and the mean movement distance of male groups (13.73 ± 8.74 km) exceeded that of females (8.95 ± 2.16 km) [57]. In contrast, the present study detected no significant sex-based difference in movement distance. This may be related to the habitat type at the release site and the provision of supplemental feeding. The Baishitougou management station in Daqingshan is situated in a montane forest ecosystem with steep terrain and multiple canyons; because of the southern fence barrier, deer could only disperse northward along the canyon networks. The deer were also cautious in their movements, even in the food-rich summer, resulting in slow exploratory dispersal. Additionally, landscape connectivity is a key factor affecting population movement, and the canyon topography is not conducive to the movement of rewilding deer [63]. Anthropogenic influences have emerged as a major selective force driving home-range plasticity in cervids. The observed contraction of winter ranges in Daqingshan Milu due to supplemental feeding parallels behavioral adaptations documented in urban white-tailed deer [64], underscoring a generalized capacity to adjust space use in response to predictable anthropogenic resources. While previous studies, such as those on Milu in the Yellow Sea tidal flats, reported expanded winter ranges during nutritionally lean months influenced by seasonal food availability and human disturbance [31], this study documented summer movement distances 7–10 times greater than in other seasons. This behavioral plasticity extends beyond simple ecological adjustments to incorporate cognitive and cultural dimensions. In migratory wapiti, for instance, spatial memory and social learning facilitate the cultural transmission of migratory routes, yet this acquired behavior remains adaptable, allowing rapid abandonment of traditional paths in response to major environmental shifts [11].

### 4.3. Crepuscular Peaks and Seasonal Plasticity in Diel Activity Rhythms of the Rewilding Milu

In this study, the diel activity rhythm of the rewilding Milu exhibited between zero (effectively resulting in zero observable cyclic patterns) and four activity peaks, with a pronounced dawn and dusk peak overall across seasons and sexes, classifying them as typical crepuscular animals. The diurnal activity rhythms of cervids typically follow a crepuscular pattern, with primary activity peaks occurring at dawn and dusk (e.g., 05:00–08:00 and 16:00–19:00), but based on species characteristics and environmental pressures they can be subdivided into three main types: crepuscular species (e.g., white-tailed deer) optimize survival by avoiding daytime heat and nocturnal predator risk [65]; roe deer shift significantly to night-time activity in areas with strong human disturbance to evade human presence [66]; and intermittent-activity species (e.g., red deer) display multiple short activity bouts throughout the day, an adaptive strategy for responding to seasonal resource fluctuations [67]. Some species, such as reindeer living in Arctic environments, even show no diel rhythmicity [68].

Human disturbance is another significant factor affecting diurnal behavioral differences in herbivores [54]. In the absence of hunting disturbance, cervid species such as white-tailed deer, moose, and roe deer all display primary activity peaks at dawn and dusk [69]. When hunting pressure increases, sika deer tend to shift their activity from a dawn-dusk pattern toward more nocturnal behavior [69]. Moose in non-tourist areas maintain natural crepuscular rhythms, but in tourist zones, their diurnal activity decreases by 70%, while nocturnal activity doubles [70]. Although the Milu in this study are located in an uninhabited zone without hunting or other human disturbances, they still exhibit noticeable monthly and seasonal differences in their diel rhythms. Many herbivores also exhibit seasonal differences in behavioral rhythms. Light and temperature are important factors in regulating circadian rhythms: large-bodied herbivores often avoid extreme heat by shifting their activities to nighttime [71]. European roe deer display a mixed nocturnal and crepuscular activity pattern in summer, rather than the typical crepuscular pattern seen in winter, as a behavioral adaptation to heat stress in high-temperature environments [72]. Based on 41 months of data, Milu showed significantly lower activity levels during the night—from midnight to 08:00—than during the day in the colder period from December to March; from April to November, their nocturnal activity remained relatively high. During the early rewilding period (October 2021–December 2021), the deer exhibited large fluctuations in diel rhythms, and the activity curves for males and females did not align, because this period represented the initial phase of adaptation. Subsequently, the diurnal activity rhythm curves for both sexes became similar, indicating that after three months the deer gradually acclimated their activity rhythms to the local environment.

## 5. Conclusions

Understanding the behavioral plasticity of rare and endangered species can provide scientific foundations for rewilding programs and improve their success rates. Under harsher conditions, rewilding Milu exhibited high behavioral flexibility in home range size, movement distance, and activity rhythms, adjusting according to season, sex, and environmental conditions. For example, during summer—when food is abundant and it is the breeding season—males showed significantly increased home ranges and movement distances, while activity timing remained focused at dawn and dusk. In contrast, during winter, when natural forage was scarce, home ranges contracted and movements became concentrated around supplementary feeding sites, reflecting food-driven aggregation. Differences in sex roles and energy strategies jointly drive these changes, complementing each other: seasonal variations in home range size driven by food availability and reproductive behavior, cautious and site-faithful movement characteristics in relation to release locations, and flexible diel rhythms—all aid Milu in optimizing foraging and risk avoidance under varying seasonal and environmental pressures. These behavioral patterns collectively reflect the strong behavioral plasticity of captive-bred Milu individuals when they are rewilding.

## Figures and Tables

**Table 1:** Basic information of GPS collared Milu in the Daqingshan Nature Reserve.

No.	Age (Years)	Sex	Monitoring Period	Valid Fixes
F1	≥5	♀	1 October 2021–30 September 2022	9831
F2	≥5	♀	1 October 2021–30 September 2022	9805
M3	3.5	♂	1 October 2021–30 September 2022	6880
F4	3.5	♀	1 October 2021–30 September 2022	8827
F5	4.5	♀	1 October 2021–30 September 2022	10,257
F6	2.5	♀	1 October 2021–30 September 2022	8724
F7	3.5	♀	1 October 2021–28 February 2025	17,228
F8	4.5	♀	1 October 2021–30 September 2022	8528
F9	3.5	♀	1 October 2021–30 September 2022	9775
M10	4.5	♂	1 October 2021–28 February 2025	16,122
M11	3.5	♂	1 October 2021–30 September 2022	4796
Total				110,733

## Data Availability

The data presented in this study are available on request from the corresponding authors.
